# Chromosomal copy number analysis of products of conception by conventional karyotyping and next‐generation sequencing

**DOI:** 10.1002/rmb2.12351

**Published:** 2020-10-08

**Authors:** Yuki Tamura, Mitsuo Santo, Yasuhisa Araki, Hidehiko Matsubayashi, Yukiko Takaya, Kotaro Kitaya, Masakazu Doshida, Kohei Yamaguchi, Shimpei Mizuta, Chie Takahashi, Namhyo Kim, Koichiro Okuno, Takumi Takeuchi, Tomomoto Ishikawa

**Affiliations:** ^1^ Nippon Reprogenetics Inc. Maebashi Japan; ^2^ Graduate School of Health Science Gunma Paz University Takasaki Japan; ^3^ Reproduction Clinic Osaka Osaka Japan; ^4^ Reproduction Clinic Tokyo Tokyo Japan

**Keywords:** chorionic villus, karyotyping, next‐generation sequencing, products of conception, spontaneous abortion

## Abstract

**Purpose:**

Chromosomal abnormalities are a major cause of spontaneous abortion, and conventional G‐banded karyotyping (G‐banding) is mainly utilized for chromosomal analysis. Recently, next‐generation sequencing (NGS) has been introduced for chromosomal analysis. Here, we aimed to investigate the applicability and utility of NGS‐based chromosomal analysis of products of conception (POC) on chorionic villus samples from spontaneous abortion.

**Methods:**

The results of chromosomal analysis of 7 chorionic villus samples from spontaneous abortion were compared between conventional G‐banding and NGS‐based chromosomal copy number analysis. Age dependency and frequency of each chromosomal aneuploidy were evaluated for 279 cases analyzed by NGS.

**Results:**

Excluding two cases (culture failure and maternal cell contamination), the results were consistent between G‐banding and NGS. For cases analyzed by NGS, the rate of chromosomal abnormality increased in a maternal age‐dependent manner. The frequency of each chromosomal aneuploidy detected by NGS was almost the same as that previously reported. Finally, NGS analysis was possible for difficult cases by G‐banding analysis, such as culture failure, maternal cell contamination, long‐term storage cases, and low cell number.

**Conclusions:**

Chromosome analysis using NGS not only obtains comparable results to conventional G‐banding, but also can analyze POC more accurately and efficiently.

## 1 INTRODUCTION

The rate of spontaneous abortion is 10%‐15% in clinically recognized pregnancies, and chromosomal abnormalities, such as aneuploidy, polyploidy, structural rearrangement, duplication, or deletion, are the most common reason.^1^, ^2^, ^3^ Conventional cytogenetic analysis by G‐banded karyotyping (G‐banding) is generally performed for chromosomal analysis, but it has several disadvantages such as low resolution, the necessity of long‐term cell culture, and enough experience for diagnosis. Moreover, cell culture failure or maternal cell contamination often occurs, which are significant obstacles for the analysis.^3^, ^4^, ^5^ Currently, molecular cytogenetic approaches, such as array comparative genome hybridization and next‐generation sequencing (NGS), have been introduced for comprehensive chromosomal analysis.^3^, ^4^, ^6^ Among them, NGS has attracted attention as an accurate, high‐resolution, and high‐throughput method and is expected to be applicable for chromosomal analysis of products of conception (POC) from spontaneous abortion.^7^, ^8^, ^9^, ^10^ In Japan, it is generally known that elderly pregnancy and late birth have been increased in association with several social factors, such as advancing of late marriage. Number of miscarriages in spontaneous pregnancy is speculated to be increased owing to the chromosomal abnormalities with advanced maternal age. In order to deal with the increasing number of chromosomal analysis of POC, more accurate and high‐throughput test methods are required such as NGS. However, to our knowledge, no reports have verified the utility of NGS‐based chromosomal analysis of POC in Japan. Therefore, in this study, we aimed to compare the consistency between conventional G‐banding and NGS‐based chromosomal copy number analysis of POC using chorionic villus samples. In addition, age dependency and frequency of each chromosomal aneuploidy were evaluated for 279 cases analyzed by NGS. Finally, points of attention for NGS‐based chromosomal analysis were discussed.

## 2 MATERIALS AND METHODS

### 2.1 Sample collection

Two studies were performed in this report, one was comparison of the consistency between conventional G‐banding and NGS‐based chromosomal copy number analysis of POC. From February 2018 to April 2018, chromosomal analysis of 7 chorionic villus samples from spontaneous abortions (7‐9 weeks) was carried out using both conventional G‐banding and NGS. Another was evaluation of age dependency and frequency of each chromosomal aneuploidy for 279 cases from February 2018 to December 2019 analyzed by NGS.

### 2.2 G‐banding and NGS

Conventional G‐banding analysis was carried out by Nihon Gene Research Laboratories Inc. For NGS‐based chromosomal copy number analysis, chorionic villi were collected from POC. To prevent maternal cell contamination, only distinct chorionic villus was collected under the stereomicroscope and maternal tissue (eg, endometrium or peripheral blood) was removed as much as possible. Genomic DNA was isolated using the QIAamp DNA Mini Kit (Qiagen GmbH) according to the manufacturer's instructions. Library construction was performed using the VeriSeq PGS kit (Illumina), and the MiSeq system (Illumina) was used for DNA sequencing following the manufacturer's protocol with minor modification. G‐banding and NGS analysis were carried out in different laboratories which were registered clinical laboratory by Japan Registered Clinical Laboratories Association. Each laboratory performed quality control internally and externally; however, G‐banding and NGS analysis were different not only quality control method but also detection principle and obtained results. Thus, we had to comprehensively consider the data quality, specificity, possibility of contamination, and so on, when obtained inconsistent test results.

### 2.3 Data analysis

Each chromosomal copy number was comprehensively estimated using the Bluefuse Multi Analysis Software (Illumina). This platform was demonstrated to have a detection performance of around 10 Mb of segmental aneuploidy and 20% of mosaicism. The range of mosaicism was defined as 20%‐80%, which complied with Preimplantation Genetic Diagnosis International Society guidelines.^11^, ^12^ Statistical analysis was performed using Fisher's exact test with the Holm correction for multiple testing, and differences were considered significant at *P* < .05.

## 3 RESULTS

Comparison of results obtained from conventional G‐banding and NGS is summarized in Table 1. NGS successfully analyzed all seven cases. Conversely, G‐banding only detected six cases; one case was undetectable because of cell growth failure. In the six cases analyzed by G‐banding, the results of five cases were consistent with the results of NGS. However, one case (Case 7) was suspected to have maternal cell contamination (G‐banding: 46,XX, NGS: 46,XY). Among the seven cases analyzed by the NGS, two cases (Cases 4 and 7) had normal male karyotype (46,XY) and five cases exhibited autosomal trisomy, implying that there were no cases suspected of maternal cell contamination.

**Table 1:** Summary results of chromosomal analysis by G‐banding and NGS

Case No.	Age (years)	Gestational age (weeks)	Development	G‐banding	NGS	Description
1	42	8	Blighted ovum	Culture failure	46,X,‐X,+11	
2	39	7	FHB+	47,XX,+13	47,XX,+13	
3	33	8	FHB+	47,XX,+22	47,XX,+22	
4	37	8	FHB+	46,XY	46,XY	9 times
5	40	7	FHB−	49,XXY,+15,+16	49,XXY,+15,+16	2 overnight
6	37	9	FHB+	47,XY,+22	47,XY,+22	
7	40	8	FHB+	MCC	46,XY	6 times, 1 mo

Note

Description shows the number of miscarriages and storage period of products of conception prior to analysis.

Abbreviations: FHB, fetal heartbeat; MCC, maternal cell contamination.

We then analyzed 279 cases by NGS. Sixty‐one (21.9%) cases were normal karyotype, 186 (66.7%) cases showed chromosomal abnormality, and 32 (11.5%) cases did not show distinct chorionic villi in POC specimens (Table 2). The 186 abnormal cases included 172 (61.6%) cases of aneuploidy (autosomal trisomy and sex chromosome aneuploidy), 8 (2.9%) cases of segmental aneuploidy (duplication and deletion) (Table S1), and 6 (2.2%) cases of mosaicism, indicating that more than half of the cases in this study were chromosomally abnormal.

**Table 2:** Summary results of NGS‐based chromosomal copy number analysis

Normal	Abnormal				Without villi	Total
	Aneuploidy	Segmental	Mosaic	Total		
61 (21.9%)	172 (61.6%)	8 (2.9%)	6 (2.2%)	186 (66.7%)	32 (11.5%)	279

Note

Number of cases and percentage of total are described about normal, abnormal, and without villi, respectively.

The sex distribution of the 61 normal cases and 186 abnormal cases is shown in Table 3. Normal cases included 39 (63.9%) XX and 22 (36.0%) XY, and abnormal cases included 84 (45.2%) XX, 86 (46.2%) XY, 13 (7.0%) X, and 3 (1.6%) XXY. Thus, there were more women (XX) than men (XY) in normal cases than in abnormal cases.

**Table 3:** Sex distribution of normal and abnormal cases

Case	XX	XY	X	XXY	Total
Normal	39 (63.9%)	22 (36.0%)	—	—	61
Abnormal	84 (45.2%)	86 (46.2%)	13 (7.0%)	3 (1.6%)	186

Note

Number of cases and percentage of total are described about each karyotype.

**Figure 1:**
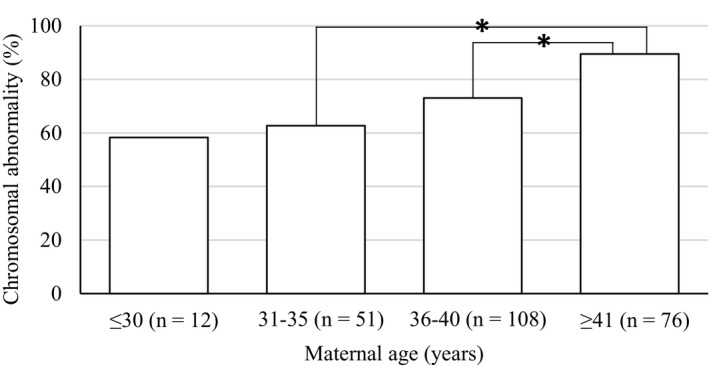
Comparison of aneuploidy rate based on the maternal age. Patients with distinct chorionic villus were divided into four groups according to maternal age (≤30, 31‐35, 36‐40 and ≥41 y), and chromosomal abnormality was estimated. Abnormal cases included segmental aneuploidies (duplication and deletion) and mosaicism. *P < .05 by Fisher's exact test with the Holm correction

Next, patients were divided into four groups according to maternal age (≤30, 31‐35, 36‐40, and ≥41 years). As shown in Figure 1, rates of chromosomal abnormality for each group were 58.3%, 62.7%, 73.1%, and 89.5%, respectively, which significantly increased in a maternal age‐dependent manner (*P* = .001).

**Figure 2:**
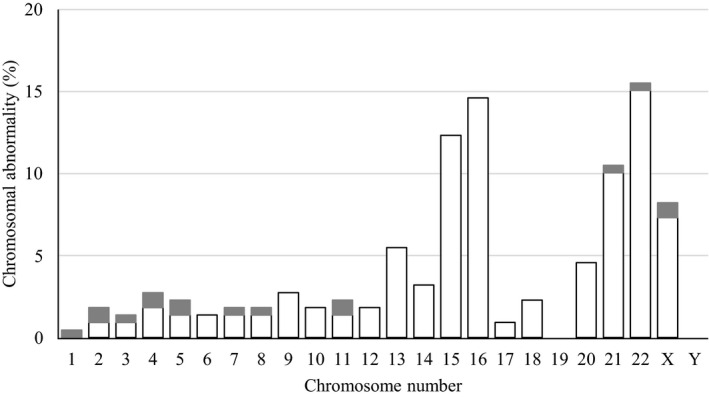
Frequency of each chromosomal aneuploidy. In 186 abnormal cases, the number of each detected chromosomal abnormality was counted, and frequency of each chromosomal aneuploidy was calculated. White bars show aneuploidy, and gray bars show segmental aneuploidies (duplication and deletion) and mosaicism

Finally, the frequency of each chromosomal aneuploidy in the 186 abnormal cases was investigated, as shown in Figure 2. Aneuploidy of chromosome 22 (34/186, 15.5%), chromosome 16 (32/186, 14.6%), chromosome 15 (27/186, 12.3%), chromosome 21 (23/186, 10.5%), and chromosome X (18/186, 8.2%) occurred most frequently among chromosomal aneuploidies.

## 4 DISCUSSION

Over 50% of miscarriages are the result of chromosomal abnormalities.^1^, ^2^, ^3^, ^4^ As described in Table 1, two cases of 46,XY were recurrent pregnancy loss cases (Cases 4 and 7), demonstrating the possibility that these abortions did not result from chromosomal abnormalities but from another reason(s) for recurrent miscarriage. The other five cases were autosomal trisomies, indicating that chromosomal abnormalities were the most common reason for spontaneous abortion. These findings are consistent with those of previous reports.^1^, ^2^, ^3^, ^4^ It has been reported that culture failure in G‐banding occurs not only due to technical problems such as minimal chorionic villi and/or prolonged time from diagnosis of miscarriage to dilation and curettage, but also because of growth arrest derived from chromosomal abnormality itself.^13^ One case of culture failure in this study was blighted ovum (Case 1); thus, NGS might be useful for difficult cases of cell culture such as specimens with few chorionic villi. In addition, NGS could analyze POC stored a long time after miscarriage, for instance, samples stored in saline at 4°C for a month (Case 7). NGS‐based analysis not only obtained equivalent results to G‐banding, but also was able to analyze specimens that were difficult to detect by G‐banding. However, additional comparative evaluations are needed due to the small number of cases included in this study.

Although villus‐like fibrous tissues could be grossly seen, sometimes tissues other than chorionic villus were observed under the stereomicroscope, as shown in Figure S1. The NGS result of these fibrous tissues indicated normal female (46,XX); therefore, maternal cell contamination was strongly suspected. Histopathologic examination suggested that most fibrous tissues were large vessels or endometrium with morphological changes caused by the action of progesterone (data not shown). In the case of blighted ovum, small or unclear fetus, and miscarriage at home, distinct chorionic villi were often not identified. Even in such cases, it is important to collect distinct chorionic villi for accurate testing, and small villi would occasionally be found in the saline used for rinsing (Figure S2). These observations suggested that the accuracy of the analysis might be improved by using tissue fragments and rinsing solution in specimens without distinct chorionic villi.

The rate of maternal cell contamination on conventional G‐banding has been reported.^5^ Lathi et al^14^ estimated the rate of maternal cell contamination on conventional G‐banding as >60% by single nucleotide polymorphism (SNP) microarray. The reported sex distribution was 57.4% XX and 42.6% XY, excluding maternal cell contamination cases. Our results were comparable with these rates (Table 3), implying that maternal cell contamination might be infrequent with NGS. However, identification of actual cell origin requires specific analysis, such as short tandem repeat analysis. Turner's syndrome (45,X) was observed in 7.0% of abnormal cases, which is similar to previous results.^2^, ^8^ Furthermore, it was well known that NGS can detect some polyploids with unbalanced sex chromosome (69,XXY and 69,XYY), but not 69,XXX and 92,XXYY.^7^ There was no case of detectable polyploids in this study; however, undetectable polyploids might be included in normal cases. Further investigation of polyploidy is needed, such as SNP microarray analysis.

Figure 1 demonstrated that the rate of chromosomal abnormality increased in a maternal age‐dependent manner; especially, about 90% of embryos derived from mothers ≥41 years were chromosomally abnormal. High frequency of chromosomal abnormality is generally appreciated as a cause of infertility and miscarriage in advanced maternal age.^2^, ^8^ The frequency of each chromosomal aneuploidy was previously investigated by several methods.^2^, ^6^, ^7^, ^8^, ^9^, ^10^ The interpretations demonstrated that chromosomes 16, 22, X, 21, and 15 tended to be frequently aneuploid, and our data are consistent with these previous results (Figure 2).

In conclusion, chromosome analysis using NGS obtained comparable results to conventional G‐banding and could detect segmental aneuploidy or mosaicism. NGS‐based analysis does not require cell culture or large cell number and is able to examine the difficult case to analyze by conventional G‐banding. These results suggest that chromosome analysis using NGS is a useful method for accurate and efficient POC testing in spontaneous abortion.

## CONFLICT OF INTEREST

The authors declare no conflicts of interest associated with this research.

## HUMAN RIGHTS AND INFORMED CONSENT

All procedures followed were in accordance with the ethical standards of the responsible committee on human experimentation (institutional and national) and with guidelines set forth by the Helsinki Declaration of 1964 and its later amendments. The study was approved by the clinical ethics committee, and informed consent was obtained from all patients for inclusion in this study.

## ANIMAL STUDIES

This study does not contain any studies with animal subjects performed by any of the authors.

## Supporting information

Figure S1

Figure S2

Table S1
