# Supplementary figures and images for: Chromosomal copy number analysis of products of conception by conventional karyotyping and next‐generation sequencing
Source: Reprod Med Biol. 2020 Oct 8;20(1):71–5. doi: 10.1002/rmb2.12351 (PMC7812460; doi:10.1002/rmb2.12351)

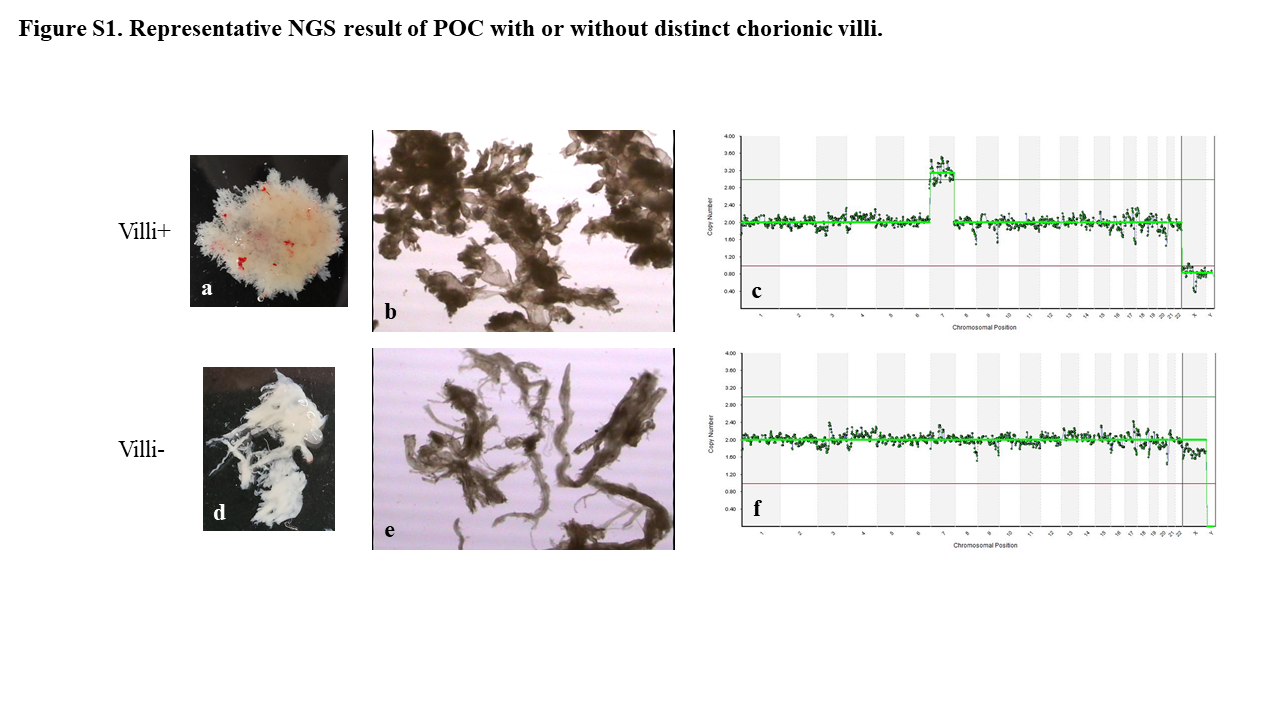

Supplement: Supplementary file 1 — Figure S1 [file RMB2-20-71-s001.TIF]

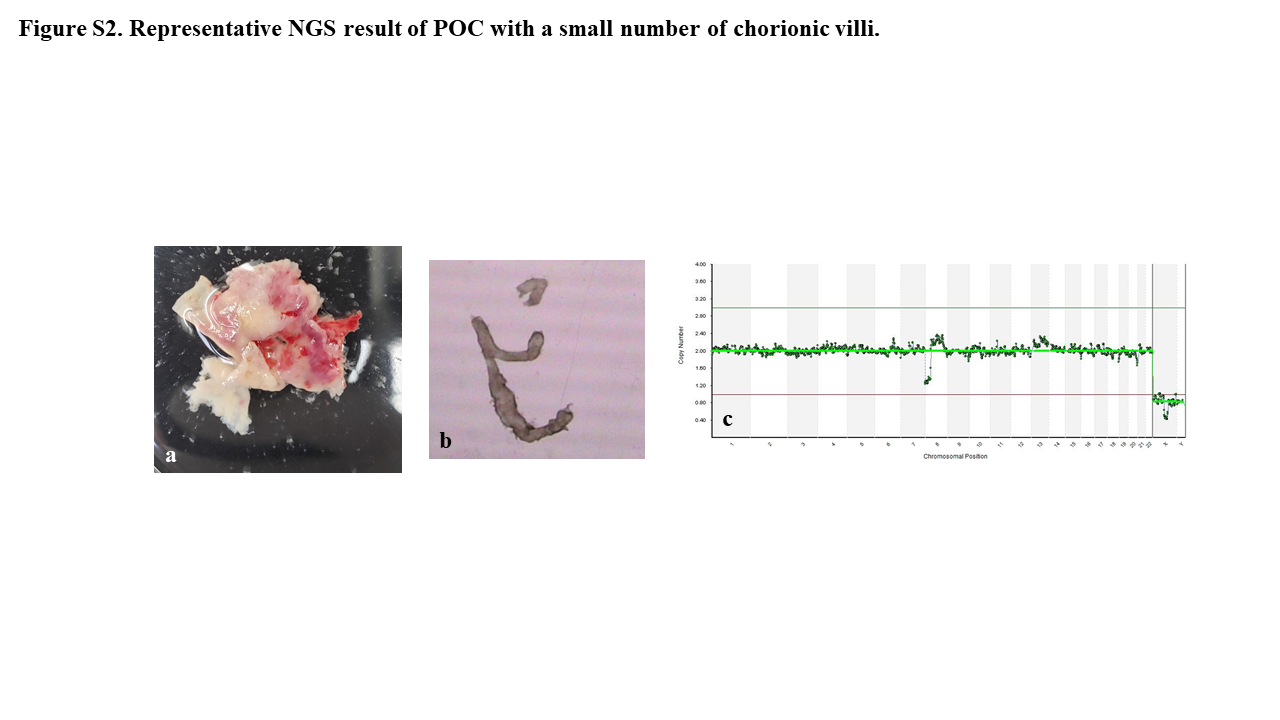

Supplement: Supplementary file 2 — Figure S2 [file RMB2-20-71-s002.TIF]
