# Supplementary material for: Chromosomal copy number analysis of products of conception by conventional karyotyping and next‐generation sequencing
Source: Reprod Med Biol. 2020 Oct 8;20(1):71–5. doi: 10.1002/rmb2.12351 (PMC7812460; doi:10.1002/rmb2.12351)
Supplement: Supplementary file 3 — Table S1 [file RMB2-20-71-s003.pdf]

Table S1. Chromosome structural information of duplication/deletion cases.

**Case No.**	**Copy number variations (CNVs)**	**Size**
1	dup(1)(q12q44), del(4)(p16.3p15.32)	115.7Mb, 15.6Mb
2	dup(7)(p22.3q11.21)	6.5Mb
3	del(5)(p15.33p15.1), dup(5)(p15.1p11)	1.7Mb, 29.8Mb
4	dup(3)(p12.3q29)	119.2Mb
5	del(11)(q13.4q23.2)	38.3Mb
6	dup(21)(p11.1q21.1)	2.3Mb
7	del(8)(p23.3p11.23)	3.7Mb
8	dup(4)(q31.3q34.1), del(4)(q34.1q35.2)	21.0Mb, 17.2Mb

Chromosome structural information (CNVs and size) of eight duplication/deletion cases are demonstrated.
